# Establishment and drug resistance characterization of paired organoids using human primary colorectal cancer and matched tumor deposit specimens

**DOI:** 10.1007/s13577-024-01139-x

**Published:** 2024-11-04

**Authors:** Jiao Deng, Jerry H. Qin, Xiaolan Li, Deding Tao, Yongdong Feng

**Affiliations:** 1 Molecular Medicine Center, Tongji Hospital, Tongji Medical College, Huazhong University of Science and Technology, 1095 Jiefang Ave, Wuhan, 430030 China; 2 Department of Surgery, Tongji Hospital, Tongji Medical College, Huazhong University of Science and Technology, 1095 Jiefang Ave, Wuhan, 430030 China; 3 Wuhan Britain-China Senior High School, Wuhan, 430030 China

**Keywords:** Colorectal cancer, Organoid, Primary tumor, TD, Drug resistance

## Abstract

Tumor deposits (TDs) represent a specific form tumor metastasis observed in colorectal cancer (CRC). The lack of successfully established cell lines for TDs, as well as the molecular mechanisms by which TDs occur remain largely unknown. Here, we established paired CRC organoids, including a human primary cancer organoid and its TD organoid, from a 46-year-old male patient with CRC. Further analysis revealed that, compared with primary tumor-derived cells, TD-derived cells exhibited enhanced proliferative, invasive and metastatic capabilities, and increased expression of stemness-related proteins. Furthermore, the present findings also demonstrated that TD-derived cells were more resistant to oxaliplatin or 5-FU. Transcriptomic profiling and qPCR revealed that TD-derived cells exhibited more alterations in fatty acid metabolism signaling and enhanced lipid synthesis ability compared to primary tumor-derived cells. Inhibition of lipid synthesis markedly decreased resistance to oxaliplatin in TD-derived cells. Taken together, the paired organoids established using CRC primary tumor and its TD specimens will provide valuable tools to study tumorigenicity, metastasis and chemoresistance in CRC. Notably, these models will provide novel insights to study tumor heterogeneity and lipid metabolism in CRC.

**Supplementary Information:**

The online version contains supplementary material available at 10.1007/s13577-024-01139-x.

## Introduction

While incidence and mortality rates have decreased in recent decades due to improved screening and treatment, colorectal cancer (CRC) remains the second most diagnosed cancer and the second leading cause of cancer deaths worldwide [1]. Cancer-related death is caused primarily by tumor metastasis [2, 3]. Despite the increasing advances in treatment, metastatic CRC-induced mortality remains high among cancer-related deaths [4, 5]. Thus, understanding the mechanisms of metastasis may provide insights for improving the survival of patients with CRC.

Tumor deposits (TDs) are one type of tumor metastasis in CRC, and these are defined as focal aggregates of cancer cells discontinuous from the primary tumor and independent of lymph nodes, vessels or nerve tissue [6]. TDs are detected in ~ 20% of patients with CRC and have been demonstrated to be associated with poor prognosis in several cohort studies [7]. However, the molecular mechanism underlying the development of TDs and their cell-of-origin remain largely unknown.

Organoids are one of the cutting-edge models in cancer research, allowing tumor-related studies to be performed both in vivo and in vitro [8–12]. Recent studies have demonstrated that paired organoids derived from primary and matched liver metastatic tumors of patients with CRC could be used to model cancer metastasis and effectively predict chemotherapy response and clinical prognosis [13, 14]. At present, established TD-derived organoids or cell lines are rare, and importantly, paired organoids derived from a primary tumor and matched TD in CRC are lacking. To uncover the underlying genetic and molecular mechanisms in the development of TDs and elucidate their cell-of-origin, establishment of paired patient-derived organoids (PDOs) from a primary tumor and matched TD in CRC is urgently needed.

The study successfully established paired PDOs, 45P and 45E, originating from a primary tumor and its TD from a Chinese male patient with CRC. The present study demonstrated that 45E possessed higher proliferative and migratory capacity and chemoresistance, preferentially expressed stem cell genes compared to 45P. Furthermore, 45E preferentially underwent lipid synthesis and became less chemoresistant when lipid synthesis was inhibited. Overall, the present study presents this pair of newly established PDOs, which may be useful tools to study chemoresistance and therapeutic targets, particularly in patients with CRC with TD.

## Materials and methods

### Cell lines and cell culturing

Human MDA-MB-231 and LoVo cells were purchased from the American Type Culture Collection (ATCC), MDA-MB-231 cells were maintained in L-15 medium (cat.no. KGL1801-500; KeyGEN BioTECH). LoVo cells were cultured in DMEM basal medium. FBS (cat.no.12484028; Gibco, Thermo Fisher Scientific) was added to the DMEM and L-15 basal culture medium. Cells were cultured at 37 °C in a 5% (v/v) CO2 incubator.

### Specimen collection

The surgical tumor tissue specimens employed in this investigation were ethically sourced with adherence to the principles of informed consent. Written informed consent was obtained with written informed consent from a 46-year-old male Chinese patient with ascending colon cancer, who received right hemicolectomy at Tongji Hospital (Wuhan, China). The primary tumor measured 5.5 × 4 × 3 cm. Additionally, a 0.9 × 0.8 × 0.6-cm tumor nodule was located within the mesentery of the ascending colon and 3 cm away from the primary lesion. The patient was staged as T3N1M0 according to the TNM classification system, with the primary tumor histologically characterized as moderately differentiated adenocarcinoma during post-surgical pathological analysis.

### Preparation of the single cell suspension

The procurement of fresh tumor specimens was initiated by a sequential washing procedure. Fresh tumor specimens were washed with 70% ethanol, followed by sterile PBS with an antibiotic (cat.no. 15240062; Gibco; Thermo Fisher Scientific) component. Subsequently, the tissues were sectioned into diminutive fragments using sterile scissors. These fragments were then enzymatically digested by incubation in serum-free DMEM/F12 (cat. no. 11320033; Gibco; Thermo Fisher Scientific) containing 1.5 mg/ml collagenase IV (cat. no. 17104019; Gibco; Thermo Fisher Scientific) and 20 μg/ml hyaluronidase (cat. no. H1115000; Sigma-Aldrich; Merck KGaA), and supplemented with 1X antibiotic/antimycotic (cat. no. 15240–062; Gibco; Thermo Fisher Scientific) at 37 °C for 1–2 h. The cells were incubated in red blood cell lysis buffer (cat. no. 00–4333-57; eBioscience; Thermo Fisher Scientific) on ice for 10 min to eliminate red blood cells. The isolated primary CRC cells were washed twice in PBS and resuspended for subsequent organoid culture establishment.

### Organoid culture

For organoid establishment, freshly isolated primary CRC cells were resuspended in Matrigel (growth factor reduced, phenol-free; cat. no. 354263; Corning) and seeded into 24-well culture plates at the indicated density (1,000 cells/30 µl Matrigel/well). After Matrigel aggregation, cells were covered with human colorectal organoid culture medium and incubated at 37˚C in an incubator with 5% CO_2_. The composition of the human CRC organoid culture medium was as follows: DMEM/F12 (cat. no. 11320033; Gibco; Thermo Fisher Scientific) supplemented with 1X B27 supplement (cat. no. 17504044; Gibco; Thermo Fisher Scientific), 50 ng/ml recombinant human epidermal growth factor (EGF) (cat. no. 100–47; PeproTech), 10 nM gastrin (cat. no. G9145; Sigma-Aldrich; Merck KGaA), 500 nM A83-01 (cat. no. HY-10432; MedChemExpress), 1X N2 (cat. no. A1370701; Gibco; Thermo Fisher Scientific, Inc.), 50 ng/ml recombinant human fibroblast growth factor FGF (cat. no. 45033; PeproTech.), 4 mM niacinamide (cat. no. HYB0150; MedChemExpress), 10 μM Y27632 (cat. no. HY-10071; MedChemExpress), 500 ng/ml R-spondin-1 (cat. no. 12038; PeproTech) and 100 ng/ml Noggin (cat. no. 120-10C; PeproTech). The medium was changed every 3 days. For serial passages, after 10 days of culture, whole organoids were digested using TrypLE™ Express (cat. no. 12605010; Gibco; Thermo Fisher Scientific) at 37˚C for 10 min. Individual organoid-derived cells were resuspended in PBS and used for a new round of organoid culture in Matrigel.

### Animal experiments

The execution of animal studies adhered to protocols and guidelines ratified by the Institutional Animal Care and Use Committee of Huazhong University of Science and Technology (Wuhan, China) (TJH-201901005). Briefly, 4-week-old female BALB/c nude mice were obtained from Gem Pharmatech (Jiangsu, China). The mice were randomly divided into different groups (n = 5). Organoid cells were washed twice with PBS and resuspended prior to injection. For subcutaneous xenografts, 5 × 10^5^ cells were injected per mouse. When the tumor grew to a size of 0.3 × 0.3 cm, the mice in the experimental group were injected intraperitoneally with oxaliplatin (10 mg/kg) (cat. no.09512; Sigma aldrich), and mice in the control group were injected intraperitoneally with an equal volume of saline once a week. A939572 (cat. no. HY-50709; MedChemExpress) was re-suspended in sterilized H2O vehicle at 30 mg/kg in a 50 μl dose. Mice were orally fed by using a syringe to administer the 50 μl dose once daily/mouse. The mice were euthanized by cervical dislocation 4 or 5 weeks after tumor implantation before the tumors were removed. All animal experiments were conducted in accordance with institutional guidelines and ethics approval.

### Library preparation for transcriptome sequencing and data analysis

Library construction was performed using the Illumina Truseq™ RNA sample prep kit (Illumina, Inc.) according to the manufacturer’s protocol. Briefly, total RNA was extracted and mRNA was isolated from total RNA using magnetic beads. Under the action of reverse transcriptase, using mRNA as a template for reverse synthesis of one-stranded cDNA, followed by two-stranded synthesis, forming a stable double-stranded structure. The double-stranded cDNA structure had sticky ends, which were made up to flat ends by adding End Repair Mix. Finally, the cDNA library was sequenced on an Illumina platform.

Analysis of differential gene expression between the 45P and 45E organoid lines was performed using DESeq2 software using the quantified RNA sequencing (RNA-seq) data. Differentially expressed genes (DEGs) were identified using the following thresholds: |log2 fold-change (FC)|≥ 1 and adjusted P-value < 0.05. Gene Ontology (GO) and Kyoto Encyclopedia of Genes and Genomes (KEGG) pathway enrichment analyses were conducted on DEGs using the clusterProfiler R software package. GO terms and KEGG pathways with a corrected P-value < 0.05 were considered significantly enriched. Gene set enrichment analysis (GSEA) software was additionally utilized to perform GSEA of the full RNA-seq dataset.

### Transwell, clonal culture, organoid culture and apoptosis assays

For migration assays, organoids were dissociated into single cells and 5 × 10^4^ cells were added to the upper chamber of a Transwell insert (pore size, 8 μm; cat. no. 3422; Corning). The lower chamber contained 600 μl 30% FBS as a chemoattractant. After 24 h of incubation, paraformaldehyde was used to fix the migratory cells through the insert and these were stained with crystal violet (cat. no. C8470; Beijing Solarbio Science & Technology). Images of migrating cells were captured and cells were counted at a magnification of × 100 using an inverted microscope (Nikon Corporation).

For the colony formation assay, cells were spread in 6-well plates at a density of 200 cells/well and incubated for 1 week. After 1 week, 45P and 45E were treated with oxaliplatin (1 μM),5-fluorouracil (1 μM), A939572 (10 nM) or A922500 (10 μM) (cat. no. HY-10038; MedChemExpress) for a week. The medium was removed, the colonies were fixed in paraformaldehyde and stained with crystal violet, and images were captured.

For the organoid formation assay, cells were resuspended in Matrigel (growth factor reduced, phenol-free; cat. no. 354263; Corning) and seeded into 24-well plates at the indicated density (1,000 cells/30 µl Matrigel/well). After Matrigel aggregation, cells were covered with human colorectal organoid culture medium and incubated at 37˚C in an incubator with 5% CO_2_. After 1 week, 45P and 45E were treated with oxaliplatin (1 μM) or 5-fluorouracil (1 μM). The treatment duration was 5 days to induce organoid growth inhibition. Organoid’s apoptosis analysis, oxaliplatin (10 μM), A939572 (10 nM) or A922500 (10 μM) for 24 h to induce organoid apoptosis. Images of organoids were captured at a magnification of × 100 using an inverted microscope (Nikon Corporation).

For apoptosis analysis, the organoid was cultured for ~ 7 days and the cells were treated with oxaliplatin (10 μM), 5-fluorouracil (10 μM), A939572 (10 nM) and A922500 (10 μM) for 24 h. Next, cells were collected using TrypLE™ Express (cat. no. 12605010; Gibco; Thermo Fisher Scientific) with Phenol Red (cat. no. 12605; Gibco; Thermo Fisher Scientific) and stained using the annexin V-FITC/PI kit (cat. no. 559763; BD Biosciences) according to the manufacturer’s instructions, then analyzed using flow cytometry (FACSAria; BD Biosciences).

### Western blotting

Total protein was extracted from organoids using RIPA lysis buffer with a protease inhibitor cocktail (cat. no. HY-K0010; MedChemExpress). Equal amounts of protein were separated by SDS-PAGE using 10% polyacrylamide gels. Proteins were transferred to polyvinylidene fluoride membranes (cat. no. IPVH00010; Immobilon-P), which were blocked with 5% skim milk for 1 h at room temperature. Membranes were incubated overnight at 4 °C with primary antibodies against E-cadherin (ECAD), Vimentin (VIM), snail family transcriptional repressor 2 (Slug), twist family bHLH transcription factor (TWIST), zinc finger E-box binding homeobox 1 (ZEB1), β-Actin (ACTB), CD133, Sox2, Oct4, acetyl coenzyme A carboxylase 1(ACC1), SCD1, FASN at the dilutions recommended by the manufacturers. After washing with TBS with Tween 20 (TBST) buffer three times, the membranes were incubated with horseradish peroxidase-linked secondary antibodies (dilution, 1:5,000) for 2 h at room temperature. Finally, protein bands were visualized using enhanced chemiluminescence substrate after TBST washes 3 times.

### Reverse transcription‑quantitative PCR (RT-qPCR)

Total RNA was extracted using RNA isolater reagent (cat. no. R401-01; Vazyme Biotech) and reverse transcribed using the HiScript II Q RTSuper Mix for qPCR kit (cat. no. R222; Vazyme Biotech.) according to the manufacturer's instructions. A ChamQ SYBR qPCR Master Mix kit (cat. no. Q311; Vazyme Biotech) was used to perform qPCR using an ABI 7300 Real-Time PCR System (Applied Biosystems; Thermo Fisher Scientific). qPCR results were analyzed using the comparative Ct method (2^−ΔΔCq^) with ACTB expression as an endogenous control. The primer sequences used in the present study are provided in Supplementary Table 3.

### Determination of ATP, free fatty acid, triglyceride and cholesterol content

For the quantification of ATP, different volumes of lysate were added depending on the number of organoids, and after lysis, the supernatant was centrifuged at 4˚C at 12,000 × g for 5 min and used for subsequent assays. The ATP assay was performed using a kit (cat. no. S0026; Beyotime Institute of Biotechnology) according to the manufacturer’s protocol.

For the free fatty acid measurement, organoids were lysed by ultrasonication directly in PBS then centrifuged at 8,000 rpm for 10 min at 4 °C. The supernatant was taken for the subsequent assay. Free fatty acid levels were examined using a kit (cat. no. BC0595; Beijing Solarbio Science & Technology) according to the manufacturer's protocol.

Triglyceride and cholesterol levels were determined by lysing organoids directly by ultrasonication in PBS. The lysed fluid was measured directly without centrifugation. The determination was performed using the kits (cat. nos. A111-1–1 and A110-1–1; Nanjing Jiancheng Bioengineering Institute) according to the manufacturer's protocol.

### Oil red O and BODIPY staining

To assess lipid accumulation, organoids were fixed in Oil Red O fixative, rinsed in 60% isopropanol and stained with Oil Red O solution according to the kit instructions (cat. no. G1262; Beijing Solarbio Science & Technology). After washing, nuclei were counterstained with Mayer's hematoxylin.

BODIPY 493/503 was diluted in PBS and configured as a 5 µM working solution (cat. no. MX5403; MKBio). According to the manufacturer’s protocols, organoids were incubated with BODIPY 493/503 for 30 min at room temperature, washed, counterstained with DAPI for 5 min and imaged by fluorescence microscopy.

### Short tandem repeat (STR) analysis for authentication

To validate the established organoids, STRs of 45P and 45E organoids and original tumor tissues were analyzed. STR profiles were compared with STR data of cell lines from databases such as American Type Culture Collection, RIKEN BioResource Center, DSMZ and Japanese Collection of Research Bioresources Cell Bank.

### Cell proliferation and viability assays

Cell proliferation assays were performed by adding cells (1,000 cells/well) to matrix gel, spreading them evenly in 96-well plates and incubating them in organoid medium for different durations. Cell numbers were examined using a Cell Counting Kit 8 (CCK8) assay (cat. no. HY-K0301; MedChemExpress), which was performed according to the manufacturer's protocol. The cell viability assay was performed by adding the desired drugs (10 μM oxaliplatin, 10 nM A939572 or 10 μM A922500) for 24 h according to the experimental requirements, and then the cell viability was measured using the CCK8 assay, which was performed according to the manufacturer’s protocol.

### Immunofluorescence staining of paraffin sections

Paraffin sections were dewaxed to water and repaired at high temperature. The sections were closed with a liquid containing donkey serum (10%) for 30 min. The serum on the sections was blotted up with absorbent paper, and a primary antibody 50 µl drop of pan-cytokeratin (Pan-CK) (cat. no. C2931; Sigma aldrich) (1:200) was placed on the sections. The sections were transferred to a wet box and placed in a refrigerator at 4 °C overnight. The next day, sections were brought to room temperature and incubated with fluorophore-conjugated secondary antibody (594 donkey anti-mouse IgG; 1:400) (cat. no. abs20017. Absin) for 1 h protected from light. After washing, nuclei were counterstained with DAPI (1:100) (cat. no. abs47047616. Absin) for 10 min. Finally, the sections were sealed and stored in a wet box at 4 °C.

### 5-Ethynyl-2’-deoxyuridine (EdU) proliferation assay

Cells (1,000 cells/well) were added to the matrix gel, spread evenly in a 96-well plate and incubated in organoid medium. Cells were incubated with EdU (100 µl per well; diluted 1:1,000 in complete medium) for 2 h. The EdU proliferation assay (cat. no. C10310-1; Guangzhou RiboBi) was performed according to the manufacturer's protocol of the kit.

### Statistical analysis

Statistical analysis was performed using the SPSS software package (Windows version 19.0; IBM Corp.) and Prism 8 (GraphPad; Dotmatics). All measurement data are presented as the mean ± standard deviation of at least three independent experiments and were statistically analyzed using Student's t-test (two-tailed) or ANOVA. Enumeration data were analyzed using Fisher's exact test. P < 0.05 was considered to indicate a statistically significant difference.

## Results

### Establishment of paired organoids derived from the CRC primary tumor and TD

New paired CRC organoids, denoted as 45P and 45E, were successfully established using fresh specimens derived from the primary tumor and matched TD from a patient with CRC (Table S1). To confirm that the newly established organoids were not contaminated with existing cell lines and uniqueness, STR analysis was first performed. The STR profiling revealed that 45P and 45E did not correspond to any of the cells collected in public cell banks (Table 1 and Table S2). Notably, 45P and 45E were found to have loss of the Y chromosome (LOY). LOY has been observed in a variety of cancer types, and LOY has been associated with a poor prognosis in patients with cancer [15–17]. Furthermore, LOY was confirmed in the paired organoids using qPCR detecting ubiquitously transcribed tetratricopeptide repeat containing, Y-linked and lysine demethylase 5D(KDM5D) (Fig. S1A and S1B), which are two Y chromosome-specific genes, with LoVo and MDA-MB-231 cells as the positive and negative control, respectively. The paired organoid cells were successfully frozen, thawed and passaged in organoid medium for > 10 generations, implying the establishment of paired organoids derived from the primary tumor and matched TD of a patient with CRC.

**Table 1:** Results of STR analysis

Microsatellite (chromosome)	45 T	45P	45E
Amelogenin	X, Y	X	Y
TH01	7, 9.3	7, 9.3	7, 9.3
D21S11	29, 31.2	29, 31.2	29, 31.2
D5S818	12	12	12
D13S317	9, 10	9, 10	9, 10
D7S820	10, 11	10, 11	10, 11
D16S539	9, 12	9, 12	9, 12
CSF1PO	12, 14	12, 14	12, 14
vWA	17, 18	17, 18	17, 18
TPOX	8, 11	8, 11	8, 11
D12S391	17	17	17
FGA	18, 25.2	18, 25.2	18, 25.2
D2S1338	18, 22	18, 22	18, 22
D18S51	13	13	13
D8S1179	13	13	13
D3S1358	15, 18	15, 18	15, 18
D6S1043	12	12	12
PENTAE	5, 22	5	5
D19S433	14	14	14
PENTAD	9	9	9

The STR profiles of primary CRC tumor tissue (45 T), the organoids of corresponding primary tumor (45P), and the organoids of corresponding tumor deposits (45E). The 20 core STR sites and amelogenin are listed

### Paired organoids simulate the tumor heterogeneity of their corresponding tumors and possess distinct biological properties

**Fig. 1:**
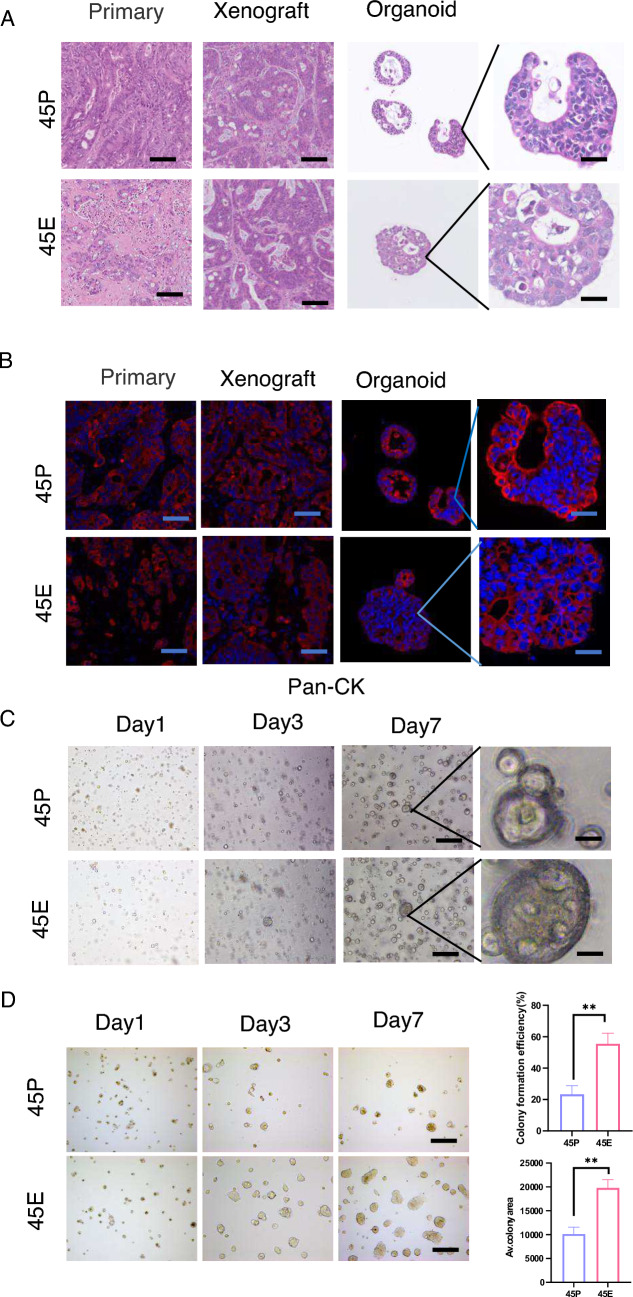
Organoids reproduce the tumor heterogeneity of CRC tumors in vitro.**A** Hematoxylin and eosin staining of the primary CRC tumor and its TD, and their corresponding xenograft and organoid. Primary and Xenograft scale bars, 100 µm; Organoid scale bars, 10 µm. **B** Immunofluorescence staining of Pan-CK in the primary CRC tumor, and the xenograft and organoid. Primary and Xenograft scale bars, 100 µm; Organoid scale bars, 10 µm. Pan-CK, pan-cytokeratin; **C** Representative images of paired organoids from primary CRC tumor cells (45P) and matched TD cells (45E) in organoid culture at different days. Left scale bars,200 µm; Right scale bars, 10 µm. **D** Representative images of clones derived from primary CRC tumor cell-generated organoids (45P) and matched TD cell-generated organoids (45E) in clonal culture at different days. The colonies were manually enumerated, and the cell area was calculated in Image J to ascertain the colony formation efficiency and mean colony area. Quantified analysis was shown. Scale bar, 200 μm. Data are presented as the mean ± SD. ^**^P < 0.05

Organoid culture is one of the main models that support long-term expansion of tumor cells in vitro [18]. To investigate whether these paired organoids simulate the tumor heterogeneity of parental tumors, H&E and Pan-CK staining of the parental tumors, paired organoids and organoid-derived xenograft tumors was conducted. The results demonstrated that the paired organoids and xenografts closely resembled their corresponding parental tumors (Fig. 1A, B). In addition, 45P and 45E cells predominantly formed spherical clusters in organoid culture, with 45P exhibiting round gland structures, while 45E exhibited complex multi-lobed morphology (Fig. 1C). Furthermore, the single cells originated from the paired organoids were able to form clones in clonal culture, and 45E-derived cells generated larger and more clones than 45P-derived cells (Fig. 1D), implying that 45E may have higher stemness. Overall, the results demonstrated that the paired organoids may simulate the tumor heterogeneity of their corresponding parental tumors and possessed distinct biological properties.

### 45E possesses higher proliferative and migratory capacity than 45P, and preferentially expresses stem cell genes

**Fig. 2:**
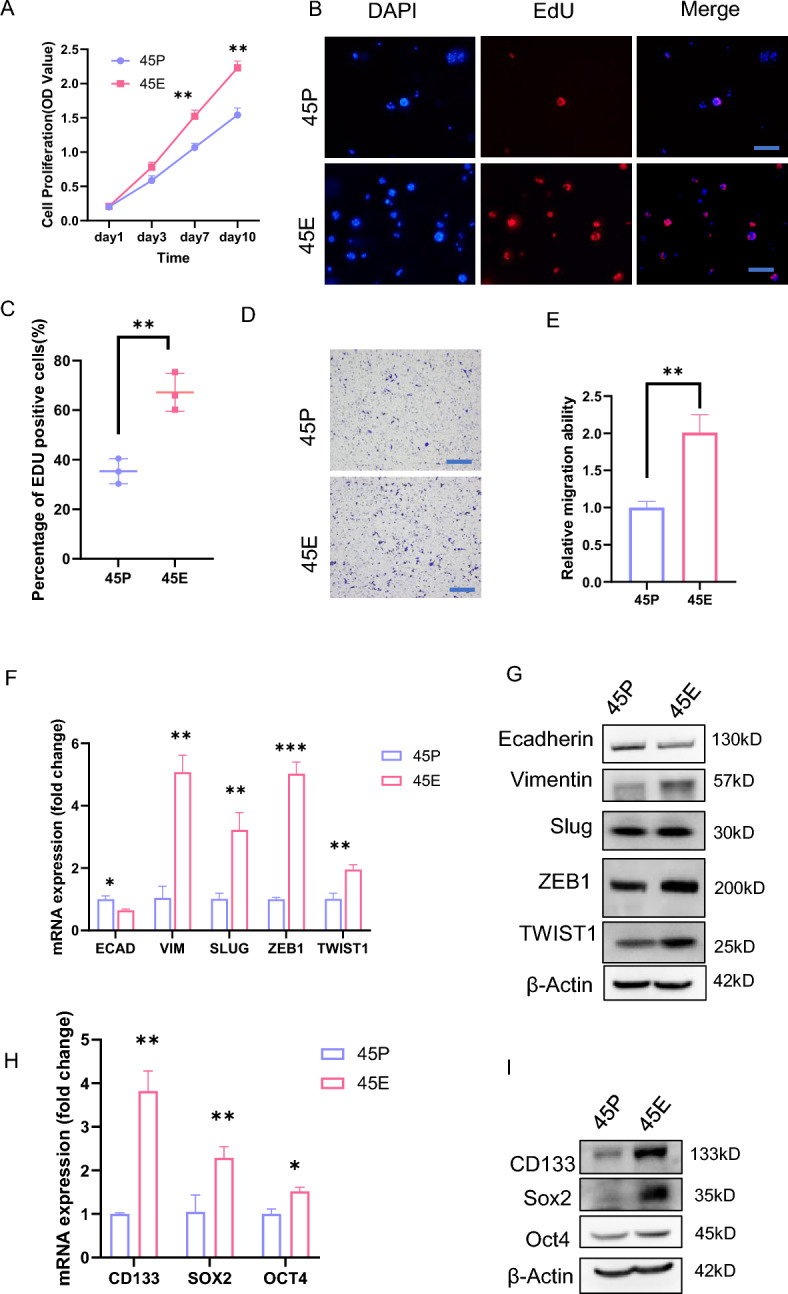
45E possesses distinct biological properties from 45P. **A** Growth curves of 45P- and 45E-derived cells based on the CCK8 assay. The experiments were repeated three times, and data are presented as the mean ± SD. ^**^P < 0.01. **B** and **C** EdU staining of 45P and 45E in EdU incorporation assays. Scale bar, 200 μm. Data are presented as the mean ± SD. Representative images were shown in **B**. Quantified analysis was shown in **C**. ^**^P < 0.01. Scale bar, 200 μm. **D** and **E** 45P- and 45E-derived cells were assessed in Transwell migration assays. Data are presented as the mean ± SD. Representative images were shown in **D**. Quantified analysis was shown in **E**. ^**^P < 0.01. Scale bar, 200 μm. **F** RT-qPCR analysis of the molecules indicated in 45P- and 45E-derived cells. Data are presented as the mean ± SD. ^***^P < 0.001, ^**^P < 0.01, ^*^P < 0.05. **G** Western blot analysis of the molecules indicated in 45P- and 45E-derived cells. (H) RT-qPCR analysis of the molecules indicated in 45P- and 45E-derived cells. Data are presented as the mean ± SD. ^**^P < 0.05, ^*^P < 0.05. **I** Western blot analysis of the molecules indicated in 45P- and 45E-derived cells

To reveal the biological properties of the paired organoids, proliferation assays, including CCK8 and EdU assays, were performed. The results demonstrated that 45E had an increased proliferative capacity compared with 45P (Fig. 2A, C). Transwell migration assays revealed that 45E cells exhibited a higher migratory capacity than 45P cells (Fig. 2D, E). To reveal the underlying mechanisms, qPCR assays were conducted in the paired organoid cells. The present findings demonstrated that 45P exhibited higher ECAD expression and lower VIM, SLUG, ZEB1 and TWIST1 expression than 45E at the mRNA and protein levels (Fig. 2F, G), implying that 45E may possess higher metastatic capacity than 45P. Since the self-renewal of tumor cells is critical for tumor development and formation of metastatic lesions [19], the expression of stem cell genes in the paired organoids was compared. The results revealed that 45E than 45P preferentially expressed stem cell genes such as CD133, SOX2 and OCT4 at the mRNA and protein levels (Fig. 2H, I). In addition, we added IHC results of 3 pairs of consecutive site tumor samples from colorectal cancer primary foci and paired TDs. IHC staining showed increased expression levels of Ki-67 and CD133, along with reduced expression levels of E-cadherin, in the TDs relative to the primary foci (Fig. S2A-S2C).

### Gene expression profiling of 45P and 45E

**Fig. 3:**
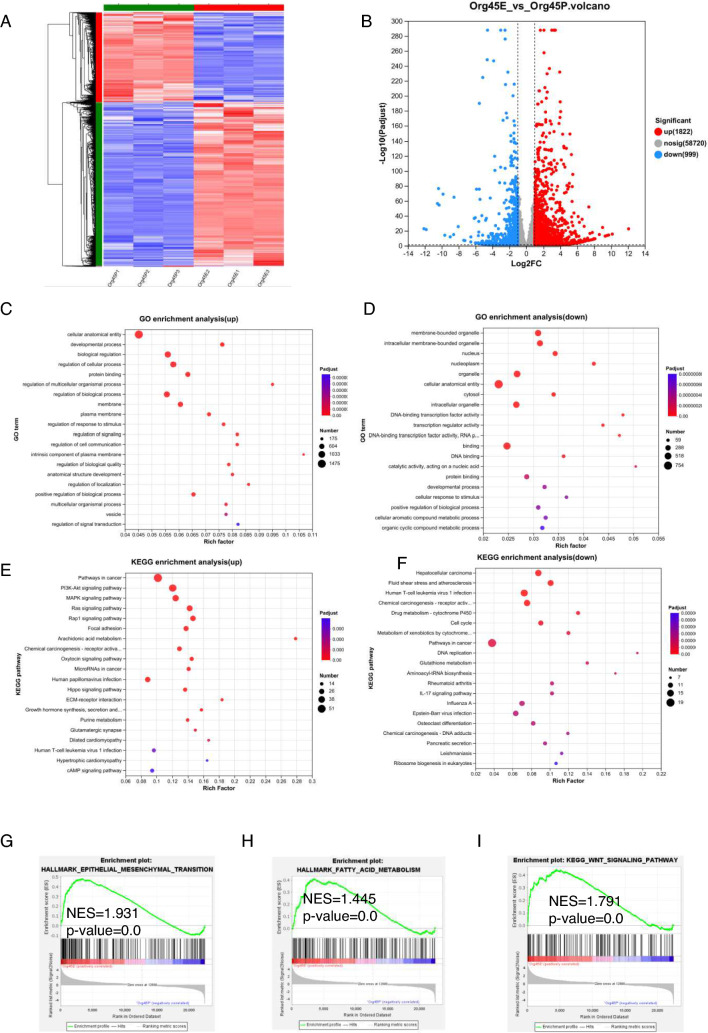
Transcriptomic profiling of 45P and 45E. **A** Heatmap presentation of hierarchical cluster analysis of DEGs. **B** Volcano plot showing DEGs. The two dashed vertical lines indicate a log_2_ fold-change of -1 (left) and 1 (right). The dashed horizontal line indicates an adjusted P-value of 0.05. **C** GO enrichment analysis results (up genes). The vertical coordinate indicates the GO term and the horizontal coordinate indicates the rich factor. The top 20 enriched biological processes were plotted in a bubble map. **D** GO enrichment analysis results (down genes). The vertical coordinate indicates the GO term and the horizontal coordinate indicates the rich factor. The top 20 enriched biological processes were plotted in a bubble map. **E**KEGG enrichment analysis results (up genes) were sorted by padjust in descending order, and the top 20 enriched pathways were plotted in a bubble map. **F** KEGG enrichment analysis results (down genes) were sorted by padjust in descending order, and the top 20 enriched pathways were plotted in a bubble map. **G** GSEA was performed and revealed the association between the gene expression of 45E-derived cells and activation of epithelial-mesenchymal transition. **H** GSEA was performed and revealed the association between the gene expression of 45E-derived cells and activation of fatty acid metabolism. **I** GSEA was performed and revealed the association between the gene expression of 45E-derived cells and activation of wnt signaling pathway. DEGs, differentially expressed genes; KEGG, Kyoto Encyclopedia of Genes and Genomes; GO, Gene Ontology; GSEA, gene set enrichment analysis; padjust, adjusted P-value

To explore the molecular basis for the different biological properties in the paired organoids, gene expression profiling was performed in 45E and 45P using RNA-seq analysis, and good replication was confirmed in triple samples and two distinct profiles using correlation analysis and principal component analysis (Fig. S3A and S3B). Post-RNA-seq data processing and normalization unveiled significantly altered gene expression profiles in 45E compared with 45P (Fig. 3A). The differential gene expression (DGE) analysis of RNA-sequencing showed that 1822 genes were up-regulated and 999 genes were down-regulated in 45E compared to 45P (fold change > 1, P value < 0.05) in 45E when compared to 45P (Fig. 3B). GO and KEGG pathway enrichment analysis put many of these DEGs into distinct functional categories. Numerous genes upregulated in 45E were involved in tumor progression, which included the MAPK signaling pathway, PI3K-Akt signaling pathway and various other oncogenic signaling pathways (Fig. 3C–F, S3C–S3F). In addition, GSEA demonstrated that epithelial-mesenchymal transition pathways and fatty acid metabolism (Fig. 3G, H), as well as additional pro-tumorigenic pathways, were preferentially activated in 45E (Fig. S3G and S3H). Furthermore, The GSEA enrichment analysis revealed that the expression profile of 45E was correlated with WNT signaling (Fig. 3I and S3I). In summary, transcriptomics coupled with bioinformatics analysis offered crucial insights into the intrinsic determinants of the biological properties distinguishing 45E from 45P.

### 45E is more resistant to chemotherapy than 45P

**Fig. 4:**
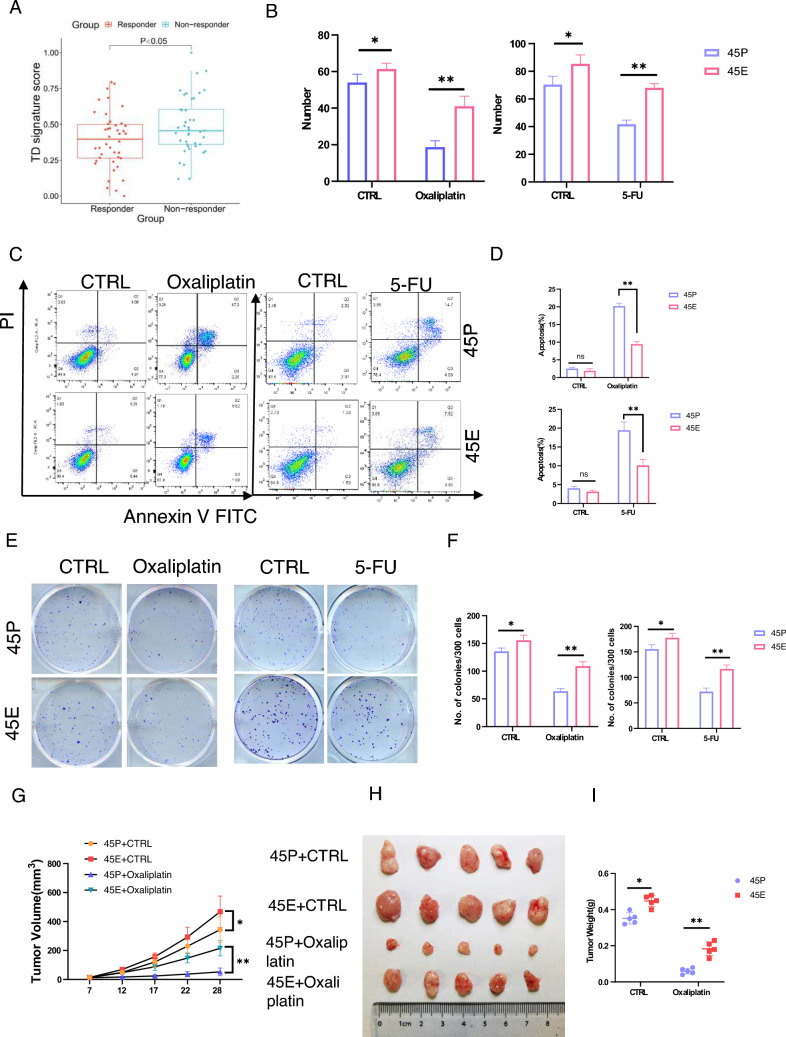
45E exhibits enhanced drug resistance compared with 45P. **A** TD gene (the top 100 up-regulated genes) signature scores are associated with chemotherapy resistance. **B** 45P- and 45E-derived cells were treated with 1 μM oxaliplatin or 1 μM 5-fluorouracil for 5 days in organoid culture. Quantified analysis was shown. Data are presented as the mean ± SD.^**^P < 0.01, ^*^P < 0.05. (C and D) 45P- and 45E-derived cells were treated with 10 μM oxaliplatin or 10 μM 5-fluorouracil in organoid culture for 24 h and then assessed by apoptosis analysis using flow cytometry. Data are presented as the mean ± SD. Representative images were shown in **C**. Quantified analysis was shown in **D**. ^**^P < 0.01. (E and F) 45P- and 45E-derived cells were treated with 1 μM oxaliplatin or 1 μM 5-fluorouracil in clonal culture for 1 week. Representative images were shown in **E**. Quantified analysis was shown in(F). Data are presented as the mean ± SD.^**^P < 0.01, ^*^P < 0.05. **G** 45P- and 45E-derived cells were subcutaneously injected into the mice (5 × 10^5^ cells per injection). At 12 days after transplantation, oxaliplatin was intraperitoneally injected into tumor-bearing mice at a dosage of 10 mg/kg once per week. Tumor volumes were calculated every 5 days and the growth curve was drawn. Tumor volumes are present as mean ± SD (n = 5);^*^P < 0.05, ^**^P < 0.01. (H and I) Four weeks after tumor implantation the mice from G were sacrificed, and the tumor weight of the subcutaneous xenografts were measured. Representative images were shown in **H**. Quantified analysis was shown in **I** Tumor weights are presented as the mean ± SD (n = 5); ^*^P < 0.05, ^**^P < 0.01

Although TDs have been demonstrated to be positively associated with poor prognosis in CRC, whether TDs lead to tumor recurrence due to chemoresistance remains unknown [20, 21]. The TD gene signature (consisting of the top 100 up-regulated genes) was established, and a validation analysis was performed using a publicly available dataset of CRC patients receiving chemotherapy (GSE28702). The results showed that the TD gene signature score was correlated with chemotherapy resistance (Fig. 4A). 45P and 45E in organoid culture were treated with oxaliplatin or 5-fluorouracil(5-FU), a commonly used chemotherapeutic agent in CRC. The results demonstrated that 45E formed larger and more organoids than 45P following administration of oxaliplatin (Fig. 4B and S5A). Following oxaliplatin or 5FU treatment, a significantly greater proportion of cells underwent apoptosis in 45P compared with 45E (Fig. 4C, D). Furthermore, 45P- and 45E-derived cells were used for the clonal culture assay in DMEM/F12 containing oxaliplatin or 5-FU. The results demonstrated that 45E-derived cells formed more clones than 45P-derived cells following treatment with oxaliplatin or 5-FU (Fig. 4E, F). These data indicated that 45E was more resistant to the chemotherapeutic agent than 45P. To determine whether 45E was more resistant to chemotherapy than 45P in vivo, 5 × 10^5^ 45P and 45E cells were injected subcutaneously into 4-week-old female BALB/c nude mice. At 14 days after transplantation, oxaliplatin was intraperitoneally injected into tumor-bearing mice at a dosage of 10 mg/kg once per week. The result showed that tumors grew faster in the 45E group than in the 45P group; meanwhile, treating oxaliplatin could slow down tumor growth in both groups (Fig. 4G). Additionally, tumors from mice with oxaliplatin treatment were significantly heavier in 45E group than in 45P group (Fig. 4H, I). Together, these findings clearly demonstrated that 45E possessed greater chemoresistance than 45P, implying that the new paired organoids may provide critical tools to uncover mechanisms of treatment failure in aggressive, oxaliplatin-resistant CRC with TDs.

### Enhanced fatty acid synthesis capacity in 45E compared with 45P

**Fig. 5:**
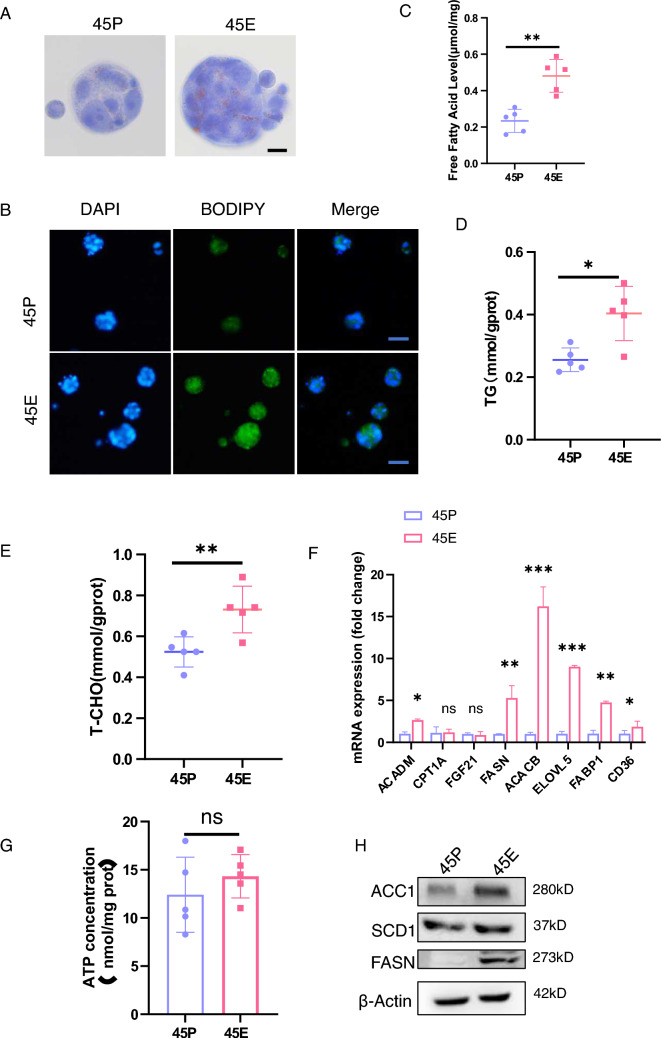
Lipid metabolism may be reprogrammed in 45E. **A** Oil red O staining of 45P- and 45E-derived cells. Representative images from experiments repeated three times Scale bar, 50 μm. **B** Representative images of BODIPY staining in 45P- and 45E-derived cells. Scale bar, 100 μm. **C** Free fatty acid levels were detected in 45P- and 45E-derived cells. Data are presented as the mean ± SD. ^**^P < 0.01. **D** Triglyceride levels were examined in 45P- and 45E-derived cells. Data are presented as the mean ± SD. ^*^P < 0.05. **E** Cholesterol levels were examined in 45P- and 45E-derived cells. Data are presented as the mean ± SD. ^**^P < 0.01. **F** Reverse transcription-quantitative PCR analysis of the molecules indicated in 45P- and 45E-derived cells. Data are presented as the mean ± SD. ^***^P < 0.001, ^**^P < 0.01, ^*^P < 0.05. **G** ATP levels were examined within 45P- and 45E-derived cells. Data are presented as the mean ± SD; ns, no significance. **H** Western blot analysis of the molecules indicated in 45P- and 45E-derived cells

Since TDs are primarily found in the pericolic or perirectal fat, we hypothesized that TDs may remodel the lipid metabolic signaling to support their growth and lead to therapy resistance. Reprogramming of lipid metabolism is mainly manifested as increased lipid synthesis, enhanced lipid oxidation and accelerated lipid translocation [22]. Previous studies have demonstrated that lipid alterations in the primary tumor and pre-metastatic ecotone may promote tumor cells to metastasize [23, 24]. Furthermore, adipocyte-derived factors may improve the survival and chemotherapy resistance of cancer cells [25]. Since GSEA demonstrated that 45E exhibited upregulated expression levels of fatty acid metabolism-related genes, the lipid droplet content in 45P vs 45E was examined by oil red O and BODIPY staining. The results revealed that 45E had larger lipid droplets than 45P (Fig. 5A, B). Intracellular free fatty acids, triglycerides and cholesterol were examined in the paired organoid cells, and the results demonstrated that 45E exhibited more fatty acids than 45P (Fig. 5C–E). To investigate the underlying mechanisms through which 45E utilizes lipid, the expression levels of enzymes in pathways such as lipid metabolism, lipid oxidative utilization lipid synthesis and lipid transport in 45E and 45P were examined using RT-qPCR. The results revealed that 45E highly expressed fatty acid synthesis (i.e., FASN, ACACB and ELOVL 5) and transport-related genes (i.e., FABP1 and CD36) at the mRNA level. However, the expression levels of fatty acid oxidation-related genes (i.e., ACADM, CPT1A and FGF21) were not significantly different between 45E and 45P (Fig. 5F). Simultaneously, an assessment of ATP levels was conducted in both 45P and 45E, revealing no statistically significant differences (Fig. 5G). Furthermore, the protein level upregulation of genes associated with fatty acid synthesis serves to substantiate the intensified lipid synthesis capacity within the 45E cells (Fig. 5H). Furthermore, we examined the expression levels of ACADM, FASN, ACC1, ELOVL5, and FABP1 in primary tumor tissues and TD tissues through IHC methods. The results showed that the expression levels of these proteins were elevated in TD tissues (Fig. S4A–S4C). Collectively, these data demonstrated that 45E reprogram lipid metabolism by enhancing lipid synthesis.

### Inhibition of lipid synthesis decreases the chemoresistance of 45E

**Fig. 6:**
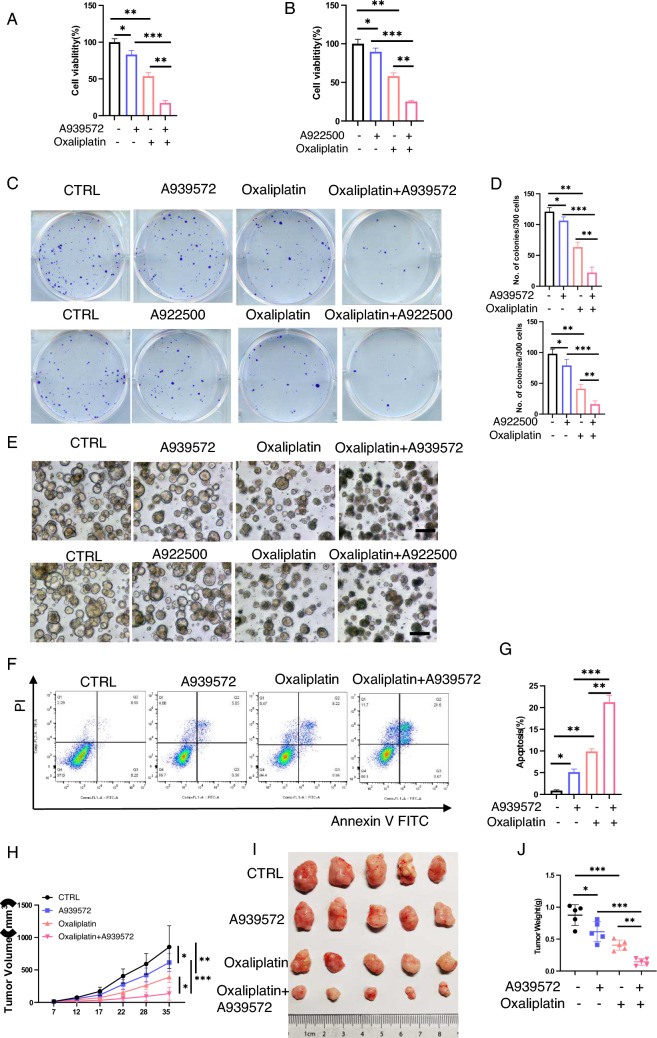
Lipid synthesis inhibitors enhance the chemosensitivity of 45E in vitro and in vivo. **A** Viability was assessed using a CCK8 assay in 45E-derived cells treated with DMSO, A939572 (10 nM), oxaliplatin (10 μM) and oxaliplatin in combination with A939572 in organoid culture for 24 h. Data are presented as the mean ± SD. ^***^P < 0.001, ^**^P < 0.01, ^*^P < 0.05. **B** Viability was assessed using a CCK8 assay in 45E-derived cells treated with DMSO, A922500 (10 μM), oxaliplatin (10 μM) and oxaliplatin in combination with A922500 in organoid culture for 24 h. Data are presented as the mean ± SD. ^***^P < 0.001, ^**^P < 0.01, ^*^P < 0.05. **C** and **D** 45E-derived cells were treated with DMSO, A939572 (10 nM) or A922500 (10 μM), oxaliplatin (1 μM) and oxaliplatin plus A939572 or A922500 in clonal culture for 1 week. Representative images were shown in (C). Quantified analysis was shown in(D). Data are presented as the mean ± SD. ^**^P < 0.01, ^*^P < 0.05. **E** 45E-derived cells were treated with DMSO, A939572 (10 nM) or A922500 (10 μM), oxaliplatin (10 μM) and oxaliplatin plus A939572 or A922500 in organoid culture for 24 h. Representative images are from triple experiments. Scale bar, 100 μm. **F** and **G** 45E-derived cells were treated with DMSO, A939572 (10 nM), oxaliplatin (10 μM) and oxaliplatin plus A939572 in organoid culture for 24 h and then assessed by apoptosis analysis using flow cytometry. Representative images were shown in **F**. Quantified analysis was shown in **G**. Data are presented as the mean ± SD. ^***^P < 0.001, ^**^P < 0.01, ^*^P < 0.05. **H** 45E-derived cells were subcutaneously injected into the mice (5 × 10^5^ cells per injection). At 12 days after transplantation, oxaliplatin was intraperitoneally injected into tumor-bearing mice at a dosage of 10 mg/kg once per week. Mice were orally fed A939572(30 mg/kg) by using a syringe to administer the 50μL dose once daily/mouse. Tumor volumes were calculated every 5 days and the growth curve was drawn. Tumor volumes are present as mean ± SD (n = 5), ***P < 0.001, **P < 0.01, *P < 0.05. **I** and **J** Five weeks after tumor implantation the mice from (H) were sacrificed, and the tumor weight of the subcutaneous xenografts were measured. Representative images were shown in **I**. Quantified analysis was shown in **J**. Tumor weights are presented as the mean ± SD (n = 5); ***P < 0.001, **P < 0.01, *P < 0.05

Lipid synthesis is an important step in metabolic pathways leading to chemotherapy resistance, and thus, tumor relapse in various types of cancer [26–28]. One study has demonstrated that inhibition of FASN and EGFR increased the sensitivity to chemotherapy in triple-negative breast cancer [29]. Since 45E exhibited enhanced fatty acid synthesis (Fig. 5F), we hypothesized that lipid synthesis inhibition may also decrease the chemoresistance to oxaliplatin in 45E. 45E cells were treated with oxaliplatin plus A939572 or A922500, two lipid synthesis inhibitors. The results indicated that 45E cells became less chemo-resistant following treatment with oxaliplatin plus lipid synthesis inhibitors compared with oxaliplatin treatment alone, as demonstrated by a marked reduction in cell viability (Fig. 6A, B). 45E cells treated with oxaliplatin or 5-FU plus lipid synthesis inhibitors such as A939572 or A922500 formed less clones compared with cells treated with oxaliplatin alone, suggesting that inhibition of lipid synthesis may decrease the chemoresistance of TD cells (Fig. 6C, D and S5B). To figure out the underlying mechanism of the decreased chemoresistance, such as increased apoptosis or decreased proliferation or both, organoid apoptosis and apoptotic assays were conducted using 45E cells treated with oxaliplatin or 5-FU plus lipid synthesis inhibitors or oxaliplatin alone. The results demonstrated that oxaliplatin plus lipid synthesis inhibitors significantly increased cell apoptosis in 45E cell (Fig. 6E–G, S5C-S5E). To explore whether 45E exhibited decreased drug resistance due to inhibited lipid synthesis in vivo 45E cells were implanted into subcutaneous of nude mice, and the mice received injection of oxaliplatin plus lipid synthesis inhibitors or oxaliplatin alone. The results showed that the tumor growth rates and weights in the oxaliplatin and A939572 treated group were significantly decreased compared with those in the control group, whereas oxaliplatin and A939572 had synergistic effects on the inhibition of tumor growth, indicating that inhibition of lipid synthesis can resist chemotherapy resistance (Fig. 6H–J). Taken together, the findings demonstrated that, inhibition of lipid synthesis, 45E were endowed with less chemoresistance, implying that combination of chemotherapeutic agents and lipid synthesis inhibitor may decrease the tumor relapse in CRCs, particularly in CRC with metastatic lesions such as TDs.

## Discussion

In the most recent 8th edition of the TNM classification for CRC, TDs have been recognized as a marker carrying prognostic implications; however, when co-occurring with lymph node metastasis, TDs did not exert an influence on tumor stage [30–33]. Recent studies havSe indicated that the presence and quantity of TDs adversely impact the prognosis of patients with tumors, irrespective of the pathological regional lymph node sub-stage. However, there have not been any novel chemotherapeutic agents proposed to specifically target TDs [34, 35]. Regarding rectal cancer, the impact of TDs is further complicated because pathological analysis typically occurs after a multidisciplinary comprehensive treatment, including chemotherapy and radiotherapy. As a result, the effect of TDs on prognosis remains a subject of controversy [36, 37]. Numerous queries persist regarding the characterization of TDs, their source, the underlying molecular mechanisms and the optimal approach to incorporate TDs into the American Joint Committee on Cancer/TNM stage.

The derivation of diverse CRC organoid models enables analysis of heterogeneous tumor characteristics [38]. Based on the commonality of the characteristics of different organoids of CRC, researchers can reveal the mechanism of colorectal cancer genesis and development and lay a theoretical foundation for the clinical treatment of CRC [39]. Previous studies have also demonstrated that primary tumors and metastatic lesions, such as liver metastases, can harbor distinct phenotypes [13, 14, 40]. Therefore, establishing organoid models from spatially distinct tumor sites is critical. The establishment of novel paired organoid lines, from both primary and metastatic lesions of the same patient, remains an important goal to capture intra-tumor heterogeneity and for clinical translational research. The present study described the establishment of 45P and 45E, a novel pair of organoids derived from a primary tumor and synchronous TD of a Chinese male patient with CRC. The experimental results suggested that 45E exhibited stronger proliferation, invasion, metastasis and stemness. Using digital spatial profiling (DSP) and shotgun proteomics, Nelleke PM Brouwer et al. found that LNMs (lymph node metastases) and TDs possessed differences in the given gene expression and signaling pathway [41]. TDs exhibited a more invasive, mesenchymal, and fibrotic phenotype, which is consistent with our findings. Our study focuses on comparing the biological characteristics of primary foci and their TD counterparts and proposing therapeutic strategies to target TDs. We have successfully cultured tumor organoid of primary foci and their paired TDs, which may serve as a valuable tool for investigating the molecular mechanisms underlying the metastasis of TDs in colorectal cancer. Molecular and functional characterization validated 45P and 45E as representative models that capture CRC heterogeneity.

The majority of tumors utilize lipids to fulfill their energy requirements, turning lipid metabolism into a pivotal pathway for generation of energy that fuels cancer cell proliferation, growth and metastasis [42, 43]. Enhanced lipogenesis, elevated lipid levels and dependence on lipid-dependent catabolism collectively contribute to tumor cells acquiring resistance to chemotherapy or targeted therapy [44, 45]. Numerous studies have demonstrated that lipid metabolism transcends a passive bystander effect within therapeutic responses [25, 46, 47]. It serves a multifaceted role by providing structural, signaling, catabolic and anabolic support, thereby cultivating a metabolic symbiotic interplay between tumor and stromal cells. Therefore, abnormal lipid metabolism has emerged as a potential therapeutic target for chemotherapy-resistant cancers. The present findings indicated that 45E TD-derived cells were more resistant to oxaliplatin than 45P primary CRC tumor-derived cells, suggesting that drug resistance of TDs may be a major factor contributing to the poor prognosis in TD-positive patients. The present data revealed notable upregulation of genes related to lipid synthesis in 45E, and the combination of oxaliplatin with lipid synthesis inhibitors displayed potential of enhancing chemotherapy response. Together, these findings elucidate mechanisms of TD-associated chemoresistance and suggest metabolism-based therapeutic strategies.

In conclusion, the results of the present study demonstrated that the paired organoid models provide a powerful system to develop precision treatment approaches for patients with CRC with TDs.

## Limitations of study

The present study provided crucial insights, there were some limitations. The organoid models were from the primary tumor and matched TD of a single patient, potentially introducing patient-specific biases. Furthermore, the mechanisms driving TD formation were not explored in-depth. To bolster the present findings, we are actively working on expanding the biobank to include paired organoids from diverse patients with CRC, encompassing varied primary tumor characteristics and genetic backgrounds. Such an expanded dataset will provide more robust tools to unveil generalizable mechanisms and therapeutic strategies relevant to patients with CRC with TDs. We performed immunohistochemical staining of tissue specimens from another three pairs of primary colorectal cancer and matched tumor deposits, but it is difficult to rule out the possibility that the findings are due to intra-tumor heterogeneity. Despite the current limitations, the present study lays a solid foundation for comprehensive exploration of the molecular underpinnings and clinical implications of TDs in the context of CRC.

## Supplementary Information

Below is the link to the electronic supplementary material.

Supplementary file1 (DOCX 666 KB)

Supplementary file2 (PDF 99 KB)

## Data Availability

All data needed to evaluate the conclusions in the paper are present in the paper and/or the Supplementary Materials. Further information and requests for resources should be directed to and will be fulfilled by the lead contact, Yongdong Feng (ydfeng@tjh.tjmu.edu.cn).
