# Supplementary figures and images for: Establishment and drug resistance characterization of paired organoids using human primary colorectal cancer and matched tumor deposit specimens
Source: Hum Cell. 2024 Nov 4;38(1):13. doi: 10.1007/s13577-024-01139-x (PMC11534897; doi:10.1007/s13577-024-01139-x)

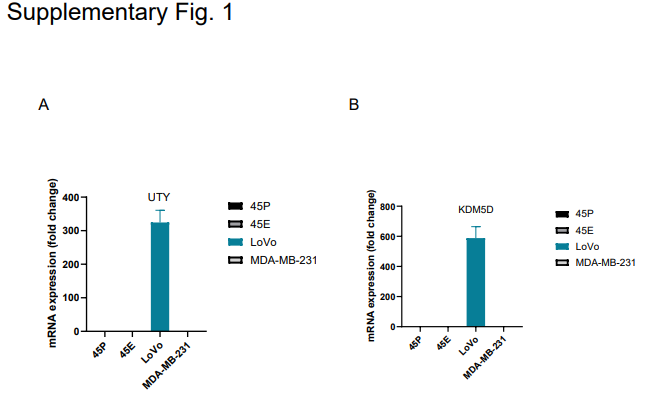


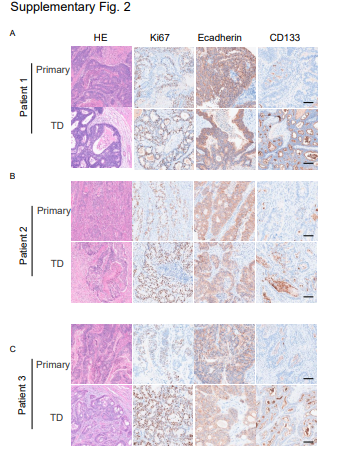


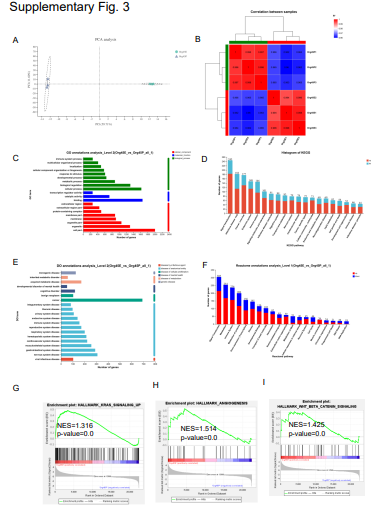


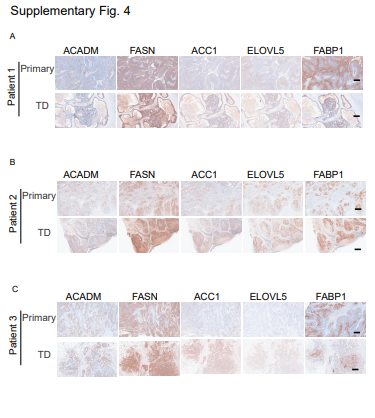


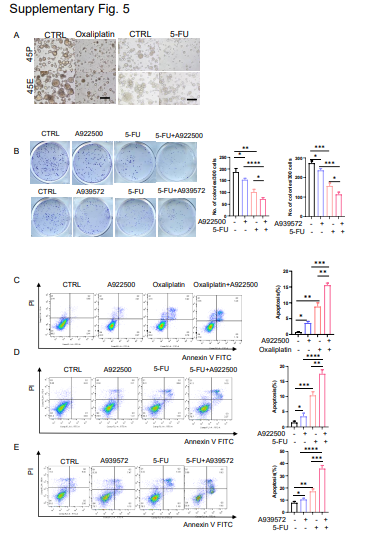

Supplement: Supplementary file 1 — Supplementary file1 (DOCX 666 KB) [file 13577_2024_1139_MOESM1_ESM.docx]
