# Supplementary material for: Establishment and drug resistance characterization of paired organoids using human primary colorectal cancer and matched tumor deposit specimens
Source: Hum Cell. 2024 Nov 4;38(1):13. doi: 10.1007/s13577-024-01139-x (PMC11534897; doi:10.1007/s13577-024-01139-x)
Supplement: Supplementary file 2 — Supplementary file2 (PDF 99 KB) [file 13577_2024_1139_MOESM2_ESM.pdf]

Table S1 Baseline characteristics of patient

Baseline characteristics of patient	
Hospital ID	1009948417
Gender	Male
Age(years)	46
BMI(kg/m2)	22.14
Tumor size(cm)	5.5*4*3
TD size(cm)	0.9*0.8*0.6
Tumor location	Ascending colon
Differentiation	Moderate
pT stage	T3
pN stage	N1
p stage	IIIb

BMI，body mass index
pT and pN stage were based on the AJCC Cancer Staging Manual,7th Edition.
pT stage:pathological primary tumor (T) stage
pN stage: pathological regional lymph nodes (N) stage
p stage: pathological stage.

Table S2 EV **Score** Comparison Details

**Sample ID**	**Sample ID**	**EV score**	**Matching specification**
45T	45P	0.933	Match
45T	45E	0.936	Match

$$\text{EV score} = \frac{\text{2xNo.shared alleles}}{\text{No.qurry alleles+No.reference alleles}}$$

Table S3 qPCR Primer List

Gene	Forward Primer	Reverse Primer
E-cadherin	CGAGAGCTACACGTTCACGG	GGGTGTCGAGGGAAAAATAGG
Vimentin	GACGCCATCAACACCGAGTT	CTTTGTCGTTGGTTAGCTGGT
SLUG	CGAACTGGACACACATACAGTG	CTGAGGATCTCTGGTTGTGGT
ZEB1	GATGATGAATGCGAGTCAGATGC	ACAGCAGTGTCTTGTTGTTGT
TWIST1	GTCCGCAGTCTTACGAGGAG	GCTTGAGGGTCTGAATCTTGCT
ACTB	CATGTACGTTGCTATCCAGGC	CTCCTTAATGTCACGCACGAT
CD133/PROM1	AGTCGGAAACTGGCAGATAGC	GGTAGTGTTGTACTGGGCCAAT
SOX2	GCCGAGTGGAAACTTTTGTCG	GGCAGCGTGTACTTATCCTTCT
OCT4	CTGGGTTGATCCTCGGACCT	CCATCGGAGTTGCTCTCCA
UTY	CGCAGTGTCGCTCACTACC	GTCAGGCTAACAGACTCCTCTT
KDM5D	CAAGACCCGCTTGGCTACATT	TTGGACGCGAGGAGTAAATCT
ACADM	ACAGGGGTTCAGACTGCTATT	TCCTCCGTTGGTTATCCACAT
CPT1A	TCCAGTTGGCTTATCGTGGTG	TCCAGAGTCCGATTGATTTTTGC
FGF21	CTGTGGGTTTCTGTGCTGG	CCGGCTTCAAGGCTTTCAG
FASN	AAGGACCTGTCTAGGTTTGATGC	TGGCTTCATAGGTGACTTCCA
ACACB	CAAGCCGATCACCAAGAGTAAA	CCCTGAGTTATCAGAGGCTGG
ELOVL5	AGTGGTGTATAACCTTGGACTCA	ACCAGAGGACACGGATAATCTTC
FABP1	ATGAGTTTCTCCGGCAAGTACC	CTCTTCCGGCAGACCGATTG
CD36	GGCTGTGACCGGAACTGTG	AGGTCTCCAACTGGCATTAGAA

Table S4 The information of patients

The information of patients					
	ID	Gender	Age	TNM stage	Number of TDs
Patient 1	60013616287	Female	76	T3N0M0	3
Patient 2	60013465875	male	81	T4N2M0	4
Patient 3	60013335977	male	58	T3N1M0	6
